# What was associated with suicide planning in middle-aged and older adults during the COVID-19 lockdown?

**DOI:** 10.1186/s13690-025-01574-8

**Published:** 2025-04-04

**Authors:** Gyeong A Kang, Ju Young Yoon, Ji Hye Shin

**Affiliations:** 1 College of Nursing, Seoul National University, Seoul, Republic of Korea; 2 Center for Human-Caring Nurse Leaders for the Future by Brain Korea 21 (BK 21) Four Project, College of Nursing, Seoul National University, Seoul, Republic of Korea; 3 Research Institute of Nursing Science, Seoul National University, Seoul, Republic of Korea

**Keywords:** Suicide plan, COVID-19, Middle-aged adults, Older adults

## Abstract

**Backgrounds:**

During the lockdown period, the challenges faced and their consequences differed by age group, making it necessary to understand the factors influencing suicidal behaviors, such as suicide planning, across different ages. This study aimed to identify the risk factors for suicide planning in middle-aged and older adults.

**Methods:**

A cross-sectional analysis was performed by combining KNHANES 2020–2021 data for people aged 40 and older with National Mental Health Statistics 2020–2021 data on the number of mental health professionals per capita. Logistic regression analysis was conducted to identify demographic, health behavior, health status, and health access factors that affected suicide planning by dividing the participants into the middle-aged (40–64 years old) and older adult (65 years old or older) groups.

**Results:**

The proportion of suicide planning among middle-aged and older adults was 1.21% and 1.36%, respectively. The proportion of participants with suicide plans who had attempted suicide was 20.52% among older adults and 14.09% among middle-aged adults. In both groups, a diagnosis of depression and high stress were consistently associated with suicide planning. In the middle-aged group, current smoking (OR = 2.27, *p* = 0.023) and unmet healthcare needs (OR = 2.32, *p* = 0.024) increased the risk of suicide planning. In the older adult group, living alone (OR = 2.72, *p* = 0.002) increased this risk.

**Conclusion:**

The prevalence of suicide attempts was higher among those with a suicide plan than among those without a suicide plan. For both middle-aged and older adult groups, it is important to provide mental health care aimed at suicide prevention, especially for those with depressive disorders or high stress levels. Additionally, providing alternative stress management resources for middle-aged smokers and monitoring isolated older adults could be effective prevention strategies.

**Table:** 

**Text box 1. Contributions to the literature**
• During the lockdown period, the challenges faced and their consequences differed by age group.
• For both middle-aged and older adult groups, it is important to provide mental health care aimed at suicide prevention, especially for those with depressive disorders or high stress levels.
• Monitoring older adults living alone may also be effective for suicide prevention.
• Providing alternative stress management resources for middle-aged smokers is suggested for suicide prevention.

## Background

Suicide is a major public health concern worldwide and is one of the leading causes of preventable deaths, with more than 700,000 people dying by suicide each year worldwide [1–3]. The suicide rate in South Korea is 22.6 per 100,000 people, the highest suicide rate among OECD countries [4]. The WHO has set a goal to reduce the global suicide rate by one-third by 2030 [3]. To prevent suicide, it is important to screen individuals at high risk of suicide [2]. In particular, the deterioration of mental health is more pronounced in disaster situations such as large-scale epidemics [5], so investigating and understanding suicide risk factors in these specific situations can provide valuable insights for future disaster preparedness.

From February 2020 to January 2022, precautionary measures were actively implemented in South Korea, including strict isolation of infected individuals and extensive public lockdowns to prevent the transmission of COVID-19 [6]. It was anticipated that sudden behavioral changes and reduced social interactions due to these measures could exacerbate feelings of isolation of individuals without support networks, potentially increasing the risk of suicide [7, 8]. However, on a global scale, suicide mortality rates did not significantly increase during COVID-19 [9]. In South Korea as well, the overall suicide rate did not increase significantly during the pandemic period [10]. Nevertheless, understanding behaviors such as suicide planning in extreme social restriction situations like lockdowns can provide a basis for prioritizing resource allocation when similar disaster situations arise in the future [8, 11]. Given the possibility of unpredictable recurrence of infectious diseases, investigating suicide risk factors during the pandemic is essential for future public health preparedness.

Suicide planning is distinct from suicidal ideation (the latter refers to the mere desire or thoughts about suicide) and is considered a significant warning sign for a suicide attempt [2]. According to a study by the WHO, only 7% of individuals with suicidal thoughts actually attempted suicide within two years [12]. Another study found that while 15.4% of individuals with suicidal thoughts but no plan attempted suicide, the rate rose to 56.0% among those who had a suicide plan [13]. Considering that many suicide attempts during lockdowns were reportedly well-planned [11], identifying the risk factors associated with suicide planning is expected to play a crucial role in preventing suicides during such periods of social restriction [1, 2].

Various factors can influence suicidal behaviors such as suicide planning and attempts [1]. In general, unmet healthcare needs, old age, mental illness, family history of suicide, socioeconomic difficulties, and adverse childhood experiences are risk factors for suicidal behavior [1, 14], and the primary risk factors may differ across age groups [15, 16]. Suicidal behavior can be triggered by stressful life events [1]. During the COVID-19 pandemic, middle-aged adults reported increased stress due to the need to readjust work and family life following the closure of schools and childcare facilities [17, 18]. Older adults were depicted in the media as particularly vulnerable to the virus, leading not only to a decline in self-esteem and perceived social worth, but also to increased social isolation [19, 20]. Considering the differing experiences among age groups during the pandemic, it is likely that the risk factors for suicidal behavior differed accordingly. Understanding the risk factors associated with suicide planning in specific age groups is crucial for establishing effective prevention strategies [7].

Pre-death events of people who had died by suicide were compared by age in a previous study, but disaster situations such as pandemics and unmet medical needs have not been considered [16]. Given the likelihood of future disaster situations, examining suicide risk factors during public lockdowns is essential for designing effective prevention strategies [8]. Therefore, this study aims to examine the factors that influenced suicide planning among middle-aged and older adults during the COVID-19 pandemic public lockdown using nationwide data.

## Methods

### Data collection and participants

#### Data source

The Korea National Health and Nutrition Examination Survey (KNHANES) is a national health survey that is conducted annually by the Korea Disease Control and Prevention Agency to assess the health and health-related behaviors of the South Korean population. To obtain the samples for KNHANES, the Population and Housing Census data was used as the sampling frame. A stratified multistage probability sampling method was used, with enumeration districts and households serving as the sampling units. Participants (about 10,000 individuals) are all family members aged 1 year or older in selected primary sampling units and households. The survey consists of a health interview, a health examination survey, and a nutrition survey. In the health survey, information such as household type, household income, etc., and information on personal health behaviors such as smoking, alcohol use, physical activity etc. is collected by face-to-face interview or by self-reporting in mobile examination centers. Sample design, subjects, survey components, and survey methods of the KNHANES are described in the Guidebook for Korea National Health and Nutrition Examination Survey database. The data and guidebook are available on KNHANES website (https://knhanes.kdca.go.kr/knhanes/main.do). Community-level data were obtained from the National Mental Health Statistics (NMHS), published annually by the Ministry of Health and Welfare and the National Center for Mental Health. NMHS is publicly available and can be accessed on the National Center for Mental Health website (www.ncmh.go.kr).

#### Study participants

**Fig. 1:**
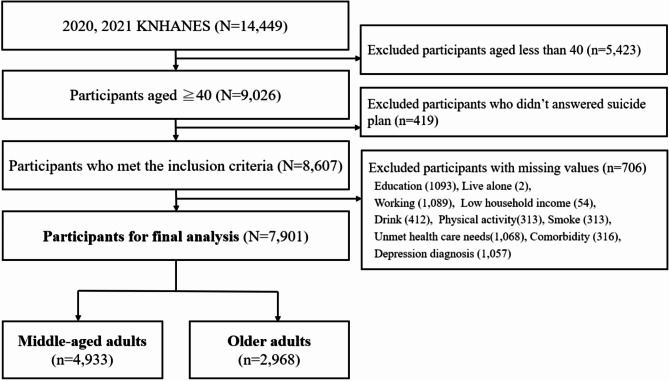
Flow chart for sample selection

We selected samples based on the KNHANES 2020 and 2021 data for people aged 40 and older, and used the combined NMHS 2020 and 2021 data to obtain information on the number of mental health professionals per capita in each administrative district. The survey included 7,359 participants in 2020 and 7,090 in 2021 (14,449 in total). Among them, we selected 8,607 participants who met the inclusion criteria: (1) aged 40 years or older, (2) answered the question about suicide planning. We excluded 706 participants with missing data for any of the variables. There is no strict definition of middle age and old age in the Korean cause-of-death statistics and a previous study [14], so the final sample (7,901 participants) was divided into middle-aged adults (aged 40 to 64 years, *n* = 4,933) and older adults (aged 65 or older, *n* = 2,968) (Fig. 1). In this study, we used 15 independent variables, so the minimum required sample size was 300, considering that an events per variable of 20 ensures model reliability [21]. The number of participants in this study was larger than the minimum required sample size, thus satisfying the participant criteria.

### Measures

#### Outcome variable: suicide plan

In the section of the KNHANES questionnaire on suicidal behaviors, participants were asked to answer the following question: “Have you seriously planned suicide in the last year?” Participants who answered “yes” were considered to have experience of suicidal planning.

#### Independent variables

Considering that participants’ behavior is influenced by social determinants [22], we classified factors potentially affecting suicide planning into sociodemographic factors, health behaviors, health status, and healthcare access.

As sociodemographic factors, we considered age (years; used as a continuous variable), gender (male vs. female), education (< 9 years vs. ≥9 years, taking into account Korea’s compulsory education system), household income level (participants were divided into the lowest-quintile group and the group that combined the other four quintiles), living alone (No vs. Yes), currently working (No vs. Yes), region (City [dong] vs. Rural [eup, myeon]), and residence in a region with the lowest quartile for the number of mental health professionals (doctors, nurses, social workers, clinical psychologists, and occupational therapists) per 100,000 population (No vs. Yes).

As health behaviors, we considered binge drinking (No vs. Yes), smoking (No vs. Yes), and low physical activity (No vs. Yes). The answer about binge drinking was considered “yes” for females if they drank 5 glasses or more of any alcohol at a time at least 2 times a week, and for males if they drank 7 glasses or more of any alcohol at a time at least 2 times a week. The answer about smoking was considered “yes” if the participants had smoked any of the following: regular cigarettes, liquid electronic cigarettes, or cigarette-type electronic cigarettes within the past month. Physical activity was used as a moderator variable and the data were obtained using the Korean version of the International Physical Activity Questionnaire Short Form (IPAQ-SF), which reportedly has high validity and reliability. Following the IPAQ scoring protocol, total minutes over the last 7 days spent on vigorous activity for work or leisure, moderate-intensity activity for work or leisure, and walking were calculated after being multiplied by 8.0, 4.0, and 3.3, respectively [23]. Physical activity < 500 METs-min/week was considered low because such activity negatively affects health outcomes [24].

As health status and healthcare access factors, we considered comorbidity (Yes vs. No) and depression diagnosis (Yes vs. No). Comorbidity was defined as having two or more of the following chronic diseases: high blood pressure, diabetes, dyslipidemia, stroke, myocardial infarction, angina, osteoarthritis, rheumatoid arthritis, asthma, kidney disease, and any cancers [25]. Considering that psychotic symptom history is primarily related to suicidal behaviors [1], the variable of doctor-diagnosed depression was used. Regarding unmet health care, the following question was asked: “during the past year, have you ever needed medical care (examination or treatment) at a hospital or clinic (excluding dentistry) but were not able to receive it?” Participants who answered “yes” were considered to have unmet health-related needs.

### Statistical analysis

KNHANES requires special statistical analysis to handle the multistage complex sampling survey design. Following the specific guidelines of KNHANES, we obtained accurate estimates and standard errors through incorporating sample weights, stratification, and clustering in the analysis. In descriptive statistics, we presented weighted means and standard errors for continuous variables, and unweighted frequencies and weighted ratios for categorical variables. To examine the factors related to suicide planning, we conducted a multivariable logistic regression analysis and calculated the odds ratios (ORs) and 95% confidence intervals (CIs).

Since the retirement age in both domestic and international contexts is typically set at 65, sociodemographic factors, health behaviors, health status, and access to healthcare services differ depending on the age group [26]. Therefore, a subgroup analysis was conducted by dividing the participants into those aged 40 to 64 years and those aged 65 years or older. The goodness-of-fit of the regression model was assessed using the Wald *F* statistics, and the explanatory power of the model was evaluated using Nagelkerke *R*². A *p*-value of less than 0.05 was considered statistically significant. Descriptive analysis and logistic regression analysis were conducted using SPSS for Windows (v.23.0; SPSS, Chicago, Illinois, USA).

### Ethics approval

This study was approved by the Institutional Review Board of Seoul National University (IRB No. E2408/001–004) and was performed in accordance with the Declaration of Helsinki.

## Results

### General characteristics

The total number of adults aged 40 and older analyzed in this study was 7,901. There were no significant differences in unmet healthcare needs and suicide plans between middle-aged and older adults. However, there were significant differences between the age groups in all other sociodemographic factors, health behaviors, and health status (Table 1).

**Table 1:** General characteristics of the sample by age group, KNHANES 2020–2021

Variables		Total (*N* = 7,901)	Middle-aged adults (*n* = 4,933)	Older adults (*n* = 2,968)	*p*
		Mean ± SE or *N* (%)	Mean ± SE or *n* (%)	Mean ± SE or *n* (%)	
*Sociodemographic factors*					
Age		57.45 ± 0.24	51.94 ± 0.15	72.57 ± 0.14	
Gender	Male	3,464 (48.59)	2,154 (50.08)	1,310 (44.5)	< 0.001
	Female	4,437 (51.41)	2,779 (49.92)	1,658 (55.5)	
Education	< 9years	2,720 (26.2)	748 (12.28)	1,972 (64.38)	< 0.001
	≥ 9years	5,181 (73.8)	4,185 (87.72)	996 (35.62)	
Annual household income	Q1–4	6,558 (87.15)	4,588 (93.98)	1,970 (68.43)	< 0.001
	Q5; Lowest	1,343 (12.85)	345 (6.02)	998 (31.57)	
Living alone	No	6,693 (87.88)	4,456 (91.49)	2,237 (78.00)	< 0.001
	Yes	1,208 (12.12)	477 (8.51)	731 (22.00)	
Working	No	3,274 (37.14)	1,470 (28.24)	1,804 (61.57)	< 0.001
	Yes	4,627 (62.86)	3,463 (71.76)	1,164 (38.43)	
Region	City	6,028 (82.21)	3,968 (84.69)	2060 (75.40)	< 0.001
	Rural	1,873 (17.79)	965 (15.31)	908 (24.60)	
Residence in a region with the lowest quartile for the number of mental health professionals	No	6061 (77.26)	3654 (75.90)	2407 (80.97)	0.004
	Yes	1840 (22.74)	1279 (24.10)	561 (19.03)	
*Health behaviors*					
Binge drinking	No	7,102 (87.83)	4,255 (85.01)	2,847 (95.55)	< 0.001
	Yes	799 (12.17)	678 (14.99)	121 (4.45)	
Smoke	No	6,616 (81.2)	3,929 (77.97)	2,687 (90.07)	< 0.001
	Yes	1,285 (18.8)	1,004 (22.03)	281 (9.93)	
Physical activity	High	2,831 (37.75)	1,969 (40.68)	862 (29.73)	< 0.001
	Low	5,070 (62.25)	2,964 (59.32)	2,106 (70.27)	
*Health status and healthcare access*					
Comorbidity	No	5,175 (70.22)	3,845 (79.37)	1,330 (45.14)	< 0.001
	Yes	2,726 (29.78)	1,088 (20.63)	1,638 (54.86)	
Depression diagnosis	No	7469 (95.05)	4694 (95.51)	2775 (93.8)	0.005
	Yes	432 (4.95)	239 (4.49)	193 (6.2)	
Stress	No	6,089 (76.4)	3,613 (73.7)	2,476 (83.9)	< 0.001
	Yes	1,812 (23.6)	1,320 (26.3)	492 (16.1)	
Unmet health care needs	No	7,351 (93.28)	4,599 (93.4)	2,752 (92.95)	0.480
	Yes	550 (6.72)	334 (6.6)	216 (7.05)	
Suicide planning	No	7,782 (98.75)	4,864 (98.79)	2,918 (98.64)	0.595
	Yes	119 (1.25)	69 (1.21)	50 (1.36)	
Suicide attempt	No	7,872 (99.76)	4,920 (99.82)	2,952 (99.62)	0.088
	Yes	29 (0.23)	13 (0.18)	16 (0.38)	

Note. The *p*-values in the univariate analyses were chi-squares (for two categorical variables). Unweighted frequency and weighted percentage. Region with a shortage of mental health professionals (in 2020: Sejong, Ulsan, Incheon, Gyeonggi; in 2021: Sejong, Ulsan, Incheon, Chungbuk)

### Distribution of suicide plans and suicide attempts

**Fig. 2:**
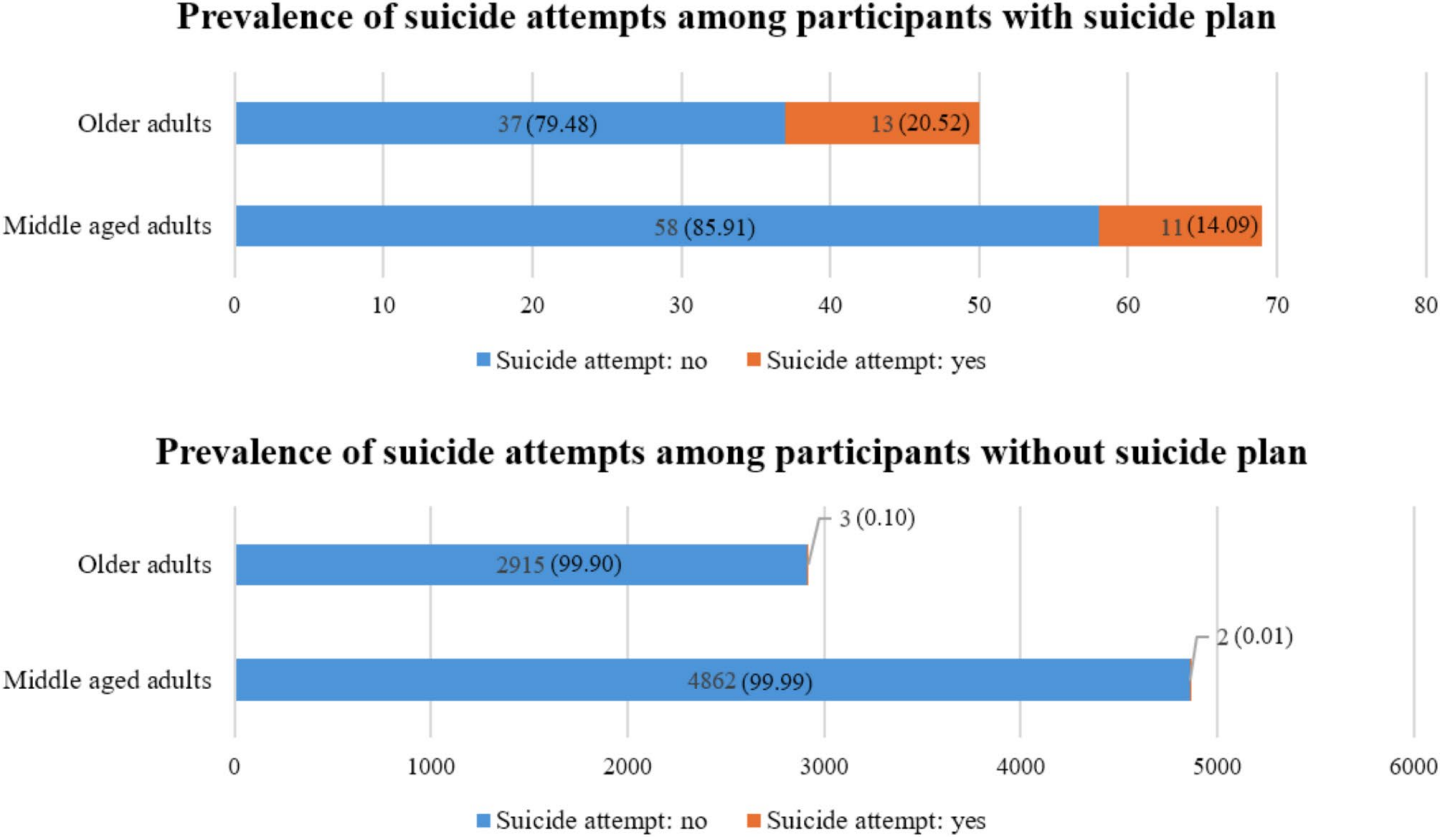
Prevalence of suicide attempts among participants with and without suicide plan

The prevalence of suicide attempts was higher among those with a suicide plan than among those without a suicide plan. Among participants with a suicide plan, 20.52% of older adults and 14.09% of middle-aged adults had a history of suicide attempts. Among participants without a suicide plan, only 0.10% of older adults and 0.01% of middle-aged adults had a history of suicide attempt (Fig. 2).

### Factors related to suicide planning

In the analysis of factors related to suicide planning, the regression model (Model 4), which included sociodemographic factors, health behaviors, health status and healthcare access, was found to be most appropriate (Table 2). The absolute values of the partial correlation coefficients among the major variables were smaller than 0.6, confirming the absence of multicollinearity. In the analysis of all participants, individuals who lived alone, were current smokers, had experienced unmet healthcare needs, had been diagnosed with depression, or reported high levels of stress were significantly more likely to have a suicide plan than those who did not have these characteristics (Table 3).

**Table 2:** Factors related to suicide planning according to multivariate analysis (*N* = 7,901)

a. Factors related to suicide planning according to multivariate analysis among middle-aged adults							
	Model 1		Model 2		Model 3		Model 4
Nagelkerke *R*^*2*^	0.046		0.069		0.204		0.215
Wald *F* (*p*-value)	5.375 (< 0.001)		6.004 (< 0.001)		14.050 (< 0.001)		13.267 (< 0.001)

b. Factors related to suicide planning according to multivariate analysis among older adults							
	Model 1		Model 2		Model 3		Model 4
Nagelkerke *R*^*2*^	0.047		0.065		0.191		0.206
Wald *F* (*p*-value)	1.936 (0.056)		2.617 (< 0.001)		7.752 (< 0.001)		6.778 (< 0.001)

Note. Sociodemographic factors: age, gender, education, annual household income, living alone, working, region, residence in a region with the lowest quartile for the number of mental health professionals; Health behaviors: binge drinking, smoke, physical activity; Health status and healthcare access factors: comorbidity, depression diagnosis, stress, unmet health care needs

Model 1 was adjusted for sociodemographic factors

Model 2 was adjusted for sociodemographic and health behavioral factors

Model 3 was adjusted for sociodemographic and health status and healthcare access factors

Model 4 was adjusted for sociodemographic, health behavioral, and health status and healthcare access factors

**Table 3:** Odds and 95% CIs from logistic regression models examining sociodemographic, health behavioral, and health status factors for suicide planning among middle aged adults and older adults (*N* = 7,901)

	Total (*N* = 7,901)			Middle-aged adults (*n* = 4,933)			Older adults (*n* = 2,968)			
	OR	95% CI	*p*-value	OR	95% CI	*p*-value	OR	95% CI	*p*-value	
Age	1.00	0.97–1.03	0.999	1.02	0.97–1.06	0.451	0.99	0.93–1.06	0.841	
Sex Male (Ref. Female)	1.20	0.66–2.15	0.549	1.23	0.57–2.66	0.594	1.54	0.56–4.22	0.402	
Education ≥ 9 years (Ref. < 9years school)	0.60	0.32–1.12	0.111	0.70	0.3–1.68	0.429	0.39	0.14–1.07	0.067	
Annual household income Q5 (Ref. Q1–4)	1.32	0.68–2.57	0.416	1.52	0.54–4.24	0.424	1.10	0.55–2.21	0.778	
Living alone Yes (Ref. No)	1.71	1.03–2.85	0.038	1.27	0.57–2.83	0.564	2.72	1.45–5.11	0.002	
Working Yes (Ref. No)	0.60	0.36-1.00	0.049	0.61	0.31–1.22	0.161	0.66	0.31–1.42	0.287	
Region City (Ref. Rural)	1.34	0.75–2.39	0.319	1.63	0.73–3.63	0.229	0.92	0.45–1.89	0.826	
Residence in a region with the lowest quantity for the number of mental health professionals Yes (Ref. No)	0.68	0.38–1.2	0.179	0.57	0.27–1.19	0.135	0.90	0.35–2.34	0.828	
Binge drinking Yes (Ref. No)	1.02	0.53–1.99	0.946	1.07	0.5–2.28	0.855	0.99	0.13–7.73	0.993	
Smoke Yes (Ref. No)	2.36	1.27–4.37	0.006	2.27	1.12–4.62	0.023	2.58	0.86–7.71	0.091	
Physical activity High (Ref. Low)	1.21	0.73-2	0.462	1.07	0.6–1.91	0.808	1.87	0.73–4.81	0.193	
Comorbidity Yes (Ref. No)	0.80	0.48–1.33	0.389	0.60	0.3–1.19	0.146	1.36	0.62–2.97	0.439	
Depression diagnosis Yes (Ref. No)	5.75	3.02–10.93	< 0.001	8.87	3.76–20.91	< 0.001	2.09	1.01–4.35	0.048	
Stress Yes (Ref. No)	5.95	3.53–10.05	< 0.001	5.01	2.56–9.78	< 0.001	9.88	4.84–20.16	< 0.001	
Unmet health care needs Yes (Ref. No)	2.04	1.15–3.63	0.015	2.32	1.12–4.83	0.024	1.67	0.72–3.86	0.233	
Nagelkerke *R*^*2*^	0.198			0.215			0.206			

### Different factors related to suicide planning among middle-aged and older adults

In the subgroup analysis of factors related to suicide planning in middle-aged and older adults, the regression model (Model 4) was found to be appropriate (middle-aged adults: Wald *F* = 13.267, *p*-value < 0.001; older adults: Wald *F* = 6.778, *p*-value < 0.001; Table 3).

Diagnosis with depression and stress were significant risk factors for suicide planning in both middle-aged and older adults (middle-aged adults: OR = 8.87, *p*-value < 0.001 for diagnosis with depression; OR = 5.01, *p*-value < 0.001 for stress; older adults: OR = 2.09, *p*-value = 0.041 for diagnosis with depression; OR = 9.88, *p*-value < 0.001 for stress; Table 3).

Among middle-aged adults, those with unmet healthcare needs were 2.32 times more likely to have a suicide plan than those without unmet healthcare needs (OR = 2.32, *p*-value = 0.024). Current smokers were 2.27 times more likely to have a suicide plan than non-smokers (OR = 2.27, *p*-value = 0.023). In the older adult group, participants who lived alone were 2.72 times more likely to have a suicide plan than those who did not live alone (OR = 2.72, *p*-value = 0.002; Table 3).

## Discussion

Using the data from the lockdown period in 2020–2021, we identified factors influencing suicide planning among middle-aged adults and older adults. After adjusting for sociodemographic, health behavior, health status, and healthcare access factors, diagnosis with depression and stress were consistently identified as significant risk factors for suicide planning in both groups. This indicates a consistent association between psychological health factors and suicide planning across life stages. Notably, suicide planning was more strongly associated with psychological health indicators than with physical health indicators in both groups. These findings were consistent with previous studies that have confirmed the relationship between psychological health and suicide planning [7, 8, 15]. For both middle-aged and older adult groups, it is important to provide mental health care to prevent suicidal behavior, especially for those with depressive disorders or high stress levels.

In this study, among participants with a suicide plan, 20.52% of older adults and 14.09% of middle-aged adults had attempted suicide. Among participants without a suicide plan, only 0.1% of older adults and 0.01% of middle-aged adults had attempted suicide. This was consistent with previous research showing that individuals with a suicide plan are more likely to attempt suicide than those without a suicide plan [13]. Research on suicide attempts is challenging due to difficulties in accessing data on individuals who have attempted or died by suicide. Therefore, prevention is key, and identifying risk factors for suicide planning, which is an important warning sign of suicide, is important for implementing preventive measures [1, 2].

Similar to a previous study, this research confirmed the heterogeneous characteristics of both age groups in terms of sociodemographic factors, health behaviors, health status, and healthcare access [26]. In this study, older adults had a higher proportion of females, lower economic activity, lower educational attainment, higher rates of living alone, more chronic diseases, and more prevalent depression diagnosis compared to middle-aged adults. Similarly, previous studies also reported a higher proportion of females among the older adults [14], as well as lower economic and educational levels and higher rates of chronic diseases compared to the middle-aged adults [27]. On the other hand, our finding regarding the rate of depression diagnosis was not consistent with the previous studies, which showed higher prevalence of depression diagnoses in middle age [28]. This discrepancy is presumably due to the differences in the causes of depression depending on age [29]. Considering the differences in sociodemographic factors across age groups, the division between middle-aged adults and older adults around the age of 65 in this study seems appropriate and reflects the characteristics of each group well.

Among all adults aged 40 and older, living alone, current smoking, unmet healthcare needs, and depression diagnosis were identified as risk factors for having a suicide plan. However, in subgroup analyses, living alone was a significant risk factor only in the older adults, while smoking was a significant risk factor only in the middle-aged adults.

Among sociodemographic factors, living alone was a significant risk factor only among older adults. While living alone does not always lead to social isolation, it is a major cause of it [30]. During the pandemic, older adults were specifically advised to stay home to limit the spread of the disease due to their vulnerability to COVID-19 [31]. Additionally, individuals at higher risk of complications from COVID-19 tended to adhere more strictly to social distancing measures [32]. Considering that older adults in this study had more comorbid chronic conditions compared to middle-aged adults, we presume that the isolation of older adults during the pandemic was further aggravated, which was a risk factor for suicide planning.

Sufficient number of care workers is generally associated with better community health outcomes [33]. However, in this study, living in one of the four regions with fewer mental health professionals per capita was not significantly related to suicide planning. This may be due to differences between administrative districts and actual living areas of people, or lower demand for mental health professionals relative to the rapid population growth [34]. Sejong City was included among cities with fewer mental health professionals per population. However, it has had the highest population growth rate among 17 provinces in South Korea—8.53% in 2019, 4.23% in 2020, and 4.40% in 2021—and also had the lowest suicide rate [34, 35].

Among health behavior factors, smoking was a significant risk factor for suicide planning only in the middle-aged adults. During the pandemic, middle-aged adults experienced increased stress due to job insecurity, financial responsibilities, and role conflicts within their households [17]. Smoking could be frequently chosen by middle-aged adults as a way to cope with stress [36]. In contrast, older adults were more likely to rely on established coping mechanisms or social support systems, even though they experienced stress due to social isolation or deteriorated health [37]. Considering these situations, providing alternative stress management resources for middle-aged adults is necessary.

Among health status factors, depression diagnosis and stress were significant risk factors for suicide planning in both middle-aged and older adults. Previous studies have shown that psychological vulnerabilities such as major depressive disorder contribute to suicidal behavior and could cause social isolation or financial insecurity [15]. In addition, drastic changes of daily life, increased economic instability, and fear regarding health during the pandemic could be stress factors [5]. To prevent further negative impacts from psychological vulnerabilities during pandemics, it is crucial to prioritize managing depression and stress among middle-aged and older adults.

Unmet healthcare needs were identified as a risk factor for suicide planning only in middle-aged adults. This was different from a previous study that found unmet healthcare needs to be a risk factor for suicide planning only in older adults [38]. Unlike Huh et al. (2024), in this study we used data exclusively from the pandemic period, so the different results may be due to varying burdens across life stages during the pandemic. In South Korea, medical services during the pandemic were focused on acute symptoms among older adults to maintain their access to healthcare services. However, middle-aged adults who had previously accessed medical services more freely experienced restrictions on healthcare access during the pandemic period [39]. In addition, middle-aged adults were at a stage of life where they may face increased caregiving responsibilities for both children and elderly parents in their family [40]. Considering that counseling services for stress or depression had been steadily increasing among people in their 40s and 50s even before the pandemic [41], reduced accessibility to existing health services could have been particularly devastating for middle-aged adults.

This study has several limitations. First, it was based on secondary data. The KNHANES dataset contains limited information on social activities, meaning that factors such as the frequency of social activities and family history of suicide could not be included. Second, this was a cross-sectional survey study, so trends or changes over time regarding suicide planning or related factors could not be examined. In future research, it is necessary to use longitudinal data to confirm trends in suicide planning and to examine relevant factors, including specific social activity information.

## Conclusion

During the pandemic, it is important to balance healthcare resources to meet the comprehensive healthcare needs of individuals [5], so effective resource allocation strategies targeting suicide prevention are urgently needed [11]. In conclusion, both middle-aged adults and older adults need prioritized management of depression and stress to prevent suicidal behavior. When considering the risk factors for suicide planning in both age groups, providing alternative stress management resources for middle-aged smokers and monitoring isolated older adults could be effective prevention strategies.

## Data Availability

All data analysed in this study is shared publicly at the KNHANES website (https://knhanes.kdca.go.kr/knhanes/main.do).

## Abbreviations

KNHANES: Korea National Health and Nutrition Examination Survey
NMHS: National Mental Health Statistics
WHO: World Health Organization
